# Autophagy: A Versatile Player in the Progression of Colorectal Cancer and Drug Resistance

**DOI:** 10.3389/fonc.2022.924290

**Published:** 2022-07-14

**Authors:** Shaista Manzoor, Jibran Sualeh Muhammad, Azzam A. Maghazachi, Qutayba Hamid

**Affiliations:** ^1^ Department of Basic Medical Sciences, College of Medicine, University of Sharjah, Sharjah, United Arab Emirates; ^2^ Department of Clinical Sciences, College of Medicine, University of Sharjah, Sharjah, United Arab Emirates; ^3^ Meakins-Christie Laboratories, Research Institute of the McGill University Health Center, Montreal, QC, Canada

**Keywords:** colorectal cancer, autophagy, epithelial-mesenchymal transition, metastasis, tumor microenvironment, therapeutic resistance

## Abstract

Colorectal cancer (CRC) is among the topmost malignancies for both genders. Despite the high incidence rate and advances in diagnostic tools, treatment in many cases is still ineffective. Most cancerous lesions in CRC begin as benign, followed by the development of invasive forms and metastases. The development of CRC has been linked to defects in autophagy, which plays both a pro-and anti-tumor role and is mainly context-dependent. Autophagy suppression could enhance apoptosis *via* p53 activation, or autophagy also promotes tumor progression by maintaining tumor growth and increasing resistance to chemotherapy. Autophagy promotes the invasion and metastasis of CRC cells *via* increased epithelial-mesenchymal transition (EMT). Moreover, dysbiosis of gut microbiota upregulated autophagy and metastasis markers. Autophagy responses may also modulate the tumor microenvironment (TME) *via* regulating the differentiation process of several innate immune cells. Treatments that promote tumor cell death by stimulating or inhibiting autophagy could be beneficial if used as an adjunct treatment, but the precise role of various autophagy-modulating drugs in CRC patients is needed to be explored. In this article, we present an overview of the autophagy process and its role in the pathogenesis and therapeutic resistance of CRC. Also, we focused on the current understanding of the role of the EMT and TME, including its relation to gut microbiota and immune cells, in autophagic manipulation of CRC. We believe that there is a potential link between autophagy, TME, EMT, and drug resistance, suggesting that further studies are needed to explore this aspect.

## Introduction

Colorectal cancer (CRC) is one of the most common malignancies in men and women, and the rate of occurrence is on the rise among young adults (1). Despite the significant attention directed at developing therapeutic and screening strategies to prevent CRC, it remains a serious disease. CRC progression from adenoma to carcinoma is a multi-stage process in which many genomic pathways play a role. At least three pathways have been identified as contributing to CRC origin and progression: Chromosomal Instability (CIN); and CpG Island Methylator Phenotype (CIMP); Microsatellite Instability (MSI). The CIN pathway is the most common cause of CRC, accounting for 85% of all. CIN-associated tumors are characterized by mutations in the *adenomatous polyposis coli* (*APC*) gene and are considered one of the earliest genetic events in colorectal cancer (2). In nearly all cases of CIN-associated tumors, Wnt signaling is activated, a crucial regulator of intestinal epithelial proliferation. CIMP is another major pathway that contributes to CRC. About 20-30% of total CRCs are CIMP-positive cancers. Most of the CIMP-related tumors are found in the proximal colon, but only 3-12% are found in the distal colon, and they are predominantly found in women. A CIMP is characterized by hypermethylation of CpG island sites that result in the inactivation of multiple cancer-related genes (3). About 15% of all CRCs demonstrate high levels of MSI. Tumors associated with the MSI pathway have defects in the MMR system due to mutations or epigenetic silencing of MMR genes, such as *MLH1, MSH2, MSH6*, or *PMS2*. This results in DNA mismatch errors within microsatellite regions. Lynch syndrome, a form of inherited cancer that accounts for three percent of all CRCs, is also associated with a germline mutation in MMR genes (*MLH1, MSH2*, and *MSH6, PMS2*) (4, 5). MMR mutations cause a hallmark phenotype known as microsatellite instability (MSI), which is characterized by changes in the length of the tandem repeat genome (6). CRCs with MSI are characterized by distinct pathological features, such as the predominance of proximal colon and/or mucinous histology as well as an increased number of lymphocytes infiltrating the tumor (7, 8). Most CRC lesions begin as benign due to mutations followed by the development of invasive forms and metastases (9). In addition to preventing these lesions from turning malignant, early detection of such benign lesions allows clinicians to halt cancer progression through therapeutic interventions (10). Currently, metastatic CRC is incurable, and the new treatment regimens focus primarily on relieving symptoms and reducing the disease progression. Therapeutic modalities for CRC include surgery, radiotherapy, chemotherapy, targeted therapy, and immunotherapy (11). Despite many advances in the availability of new diagnostic and treatments for metastatic CRC, the median survival time remains below 30 months for most patients (12). Several diseases including CRC, have been linked to defects in autophagy since its role in tumor formation is vastly complex and depends on several biological aspects. Tumorigenic and anti-tumorigenic autophagy plays a dichotomous role in cancer development. Alternative therapies like targeting autophagy may prove helpful in this regard because they can stimulate or inhibit autophagy. Subsequently, upregulation of autophagy by cancer therapies can either promote tumor cell survival or oppositely cause tumor cell death. Given this, a growing body of studies focuses on the autophagy pathway and its potential role in the pathogenesis of CRC. In the present review, we focus on the role of autophagy in the pathogenesis and therapeutic resistance of CRC.

## Introduction to Autophagy Process

**Figure 1:**
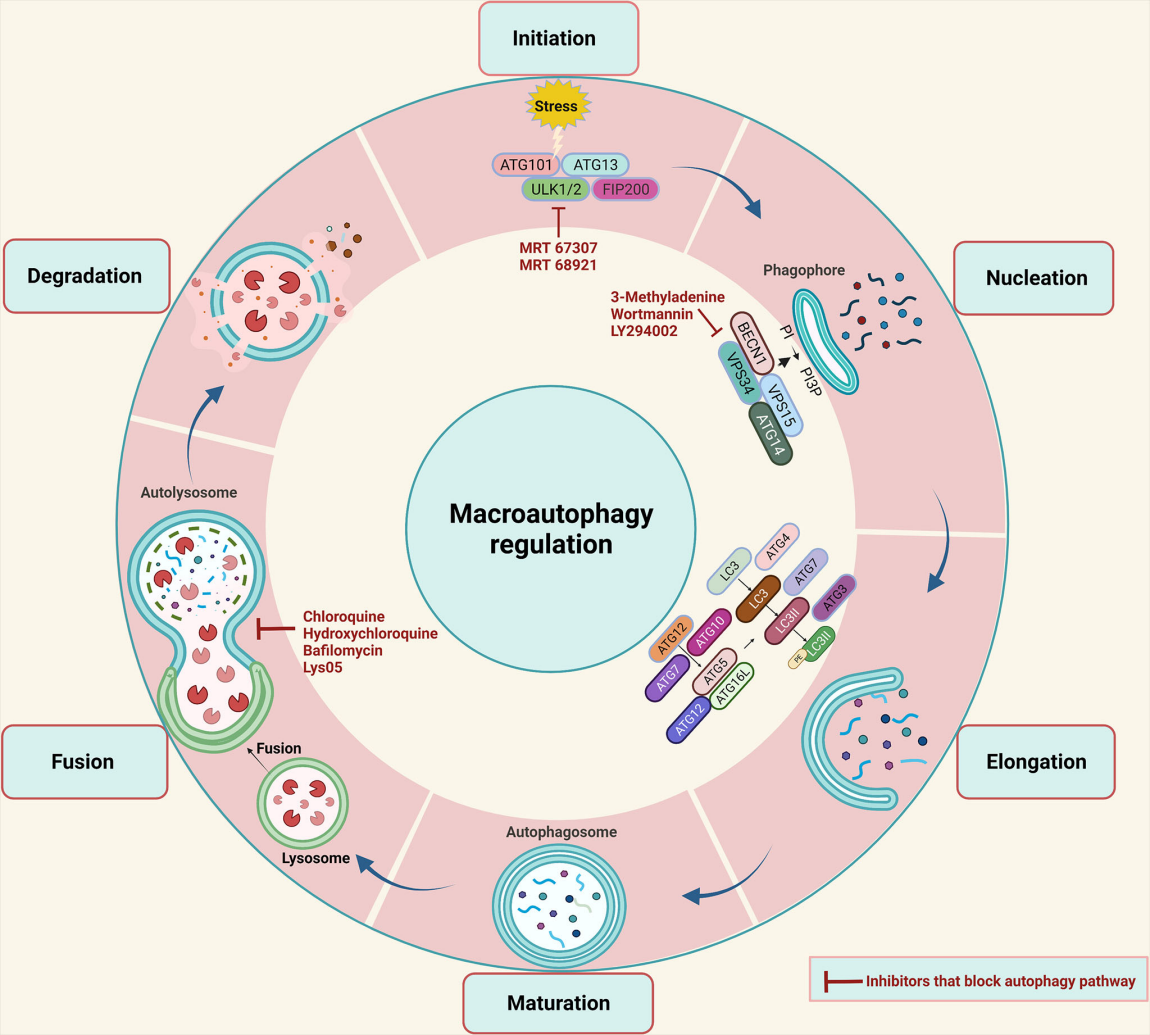
A diagrammatic representation of the macroautophagy (also known as autophagy) process is shown. A phagophore forms in autophagy that sequester cytoplasmic material in a vesicle that later matures into an autophagosome, which then fuses with lysosomes to form an autolysosome, where the sequestered material is degraded. The autophagy process takes place in several steps such as Initiation, Nucleation, Elongation, Maturation, Fusion, and Degradation. The autophagy process can be blocked at various steps with the help of autophagy inhibitors listed in the red color.

Autophagy is a physiological process that maintains metabolism and cellular homeostasis by recycling damaged cellular components. Depending on the mode of cargo delivery into the lysosomes, autophagy can be classified into three types: chaperon-mediated autophagy (CMA), microautophagy, and macroautophagy. Interestingly all autophagic pathways end up in the lysosome, but each of them differs in the regulatory mechanism and the circumstances under which it is activated. CMA occurs when a specific protein having a “KFERQ” motif is recognized by heat shock cognate 71 kDa protein (HSC70), a cytoplasmic chaperone is delivered directly to the lysosome followed by lysosomal membrane translocation through interactions with lysosomal-associated membrane protein 2 (LAMP2). To date, CMA has only been associated with aging, neurodegenerative diseases, and glioblastoma (13), but its role in CRC was not reported. In microautophagy, the lysosome directly invaginates its membrane to engulf cytoplasmic material when the cell is under starvation conditions (14). Thirdly, macroautophagy is the best-studied form of autophagy and the changes associated with it have been extensively documented in cancer research (15). Macroautophagy is a multistep process including initiation, nucleation, elongation, maturation, fusion, and degradation. In the past few years, a wide array of inhibitors of the autophagy pathway have been developed. (**Figure 1**). A family of autophagy-related (ATG) genes regulates this form of autophagy (**Table 1**).

**Table 1:** Core autophagy genes and their functions.

Core autophagy genes	Role
*ATG2*	Regulates the size of nascent autophagosomes during autophagosome assembly.
*ATG3*	Covalently binds phosphatidylethanolamine to the C-terminal glycine of ATG8-like proteins such as GABARAP, GABARAPL1, or MAP1LC3A in an E2-like manner.
*ATG4A*/*B*/*C*/*D*	Helps to reveal C- terminal glycine of ATG8 family proteins by cleaving the C-terminal amino acid.
*ATG5*	As an E3-like enzyme, ATG12-ATG5 conjugates help lipidate and bind ATG8 family proteins to vesicles.
*ATG7*	Conjugates the ATG12 protein with ATG5, and the ATG8 family proteins with phosphatidylethanolamine.
*ATG9A/B*	Functions as a key component of the preautophagosomal structure and phagophore assembly site (PAS).
*ATG10*	Conjugates ATG12 to ATG5.
*ATG12*	ATG12-ATG5 complex plays a critical role in lipidation of proteins in the ATG8.
*ATG13*	mTOR regulates autophagy by controlling the phosphorylation state of ATG13 and ULK1 and the ATG13-ULK1-RB1CC1 complex.
*ATG14*	Promotes PIK3C3 activity, contributes to the formation of autophagosomes and the conjugation of MAP1LC3/LC3 to phosphatidylethanolamine.
*ATG16L1*	Elongates the cell membrane by activating LC3 by conjugating phosphatidylethanolamine to ATG12-ATG5, resulting in a membrane-bound form of LC3 called LC3-II.
*BECN1*	It serves as a core subunit of the PI3K complex and is essential for the phosphatidylinositol-3-phosphate formation.
*C12orf44*/*Atg101*	Provides protection against proteasomal degradation of ATG13
*PIK3C3*/*VPS34*	Forms a curvature in the membrane of the endoplasmic reticulum prior to vesicle budding.
*PIK3R4*/*VPS15*	PI3K regulatory subunit involved in phosphatidylinositol 3-phosphate synthesis.
*LC3A/B/C, GABARAP, GABARAPL1/2*	Involved in the formation of autophagosome, conjugates with PE and firmly binds to the inner and outer membrane of the autophagosome.
*RB1CC1/FIP200* (functional homolog)	Regulates early and late autophagosome events by interacting with Atg16L1.
*ULK1*/*2*	Regulates the formation of autophagophores, the precursors of autophagosomes, by acting upstream of phosphatidylinositol 3-kinase PIK3C3.
*WIPI1*/*2*	Contributes to the formation of preautophagosomal structures. On endoplasmic reticulum membranes, it assists in recruiting ATG12-ATG5-ATG16L1, a complex that directly controls the elongation of the nascent autophagosome membrane.

In this process, autophagy initiation requires the translocation of the ULK1 complex consisting of ULK1, FAK family kinase interacting protein of 200 kDa (FIP200), ATG13, and ATG101 to the site of phagophore initiation, where dephosphorylation will cause the activation of this complex (16). Subsequently, the activated ULK1 complex acts as a recruitment scaffold for the class III phosphatidylinositol 3-kinase (PI3K) complex, including vacuolar protein sorting 34 (VPS34), Beclin-1, VPS15, and ATG14L-like. In response, phospholipid 3-phosphate (PI3P) is produced and is accumulated on the phagophore membrane by activated class III PI3K. This is followed by the recruitment of ATG proteins such as WD repeat domain, phosphoinositide interacting 1 (WIPI1), and WIPI2. As the phagophore elongates into a double-membrane structure, autophagosomes are formed. Two pathways of ubiquitin-like conjugation, the ATG12 conjugation system, and the ATG8 conjugation system, are essentially required for the phagophore elongation step. The ATG12 conjugation mechanism relies on ATG7 and ATG10 to help conjugate ATG12 to ATG5, which then pairs with ATG16 to produce the ATG12-ATG5-ATG16 conjugate, which is necessary for the expanding phagophore’s curvature. The ATG12-ATG5-ATG16 complex dissociates from the membrane as the autophagosome is formed, making it a suitable marker for early autophagic events (17).

Meanwhile, the ATG8 conjugation system results in the LC3 proteolytic activation. When the autophagy is initiated, LC3B is cleaved by ATG4 to form LC3B-I, which interacts with ATG7 in an ATP-dependent manner to activate LC3-I. The latter conjugates with phosphatidylethanolamine (PE) phospholipid by ATG7 and ATG3 along with the ATG12-ATG5-ATG16 complex to form LC3-II. Consequently, the membrane of the phagophore is expanded and closed. ATG8 is released from autophagosomes following elongation and maturation through deconjugation by ATG4. Afterward, autophagosomes and lysosomes merge to form an autolysosome where acidic hydrolases and lipases degrade the sequestered material to recycle it for cellular metabolism and homeostasis (18).

Several studies have suggested a complex role of autophagy in cancer development and progression. It is believed that early-stage autophagy prevents cancer development. However, when cancer has progressed, the autophagy process increases tumor cells’ ability to grow and adapt to adverse environments (19–22).

## Role of Autophagy in CRC

The autophagy pathway plays both a pro-and anti-tumor role in colon cancer and its function varies depending on biological factors such as genetic mutations, tumor type, and tumor stage (23–25). It is well known that autophagy processes can restore cellular homeostasis and prevent malignant transformation by clearing damaged organelles and foreign bodies. It also promotes tumor progression by maintaining cell stability, increasing resistance to unfavorable environments, and maintaining tumor growth (26). In CRC autophagy modulation has recently been investigated (27). **Table 2** summarizes several autophagy regulatory factors that are implicated in CRC carcinogenesis and therapeutic resistance.

**Table 2:** Pro-tumorigenic and anti-tumorigenic roles of regulatory factors of autophagy in CRC.

Regulatory factors of autophagy	Observation	Tumor response	Downstream target process/ molecule	Reference
LC3 B	Associated with aggressive CRC phenotype.	Pro-tumor	Not reported	(28)
	Associated with CRC lymphatic invasion.	Pro-tumor	Not reported	(29)
	Overexpressed in CRC compared to normal tissue	Pro-tumor	Not reported	(30)
Beclin 1	Associated with CRC aggressive phenotype	Pro-tumor	Not reported	(31)
	Associated with poor survival rate after chemotherapy in CRC	Pro-tumor	Not reported	(32)
ATG5	Tumor growth is inhibited by ATG5 inhibition in patient-derived *in vitro* models of CRC	Pro-tumor	Not reported	(33)
LAMP 3	Overexpressed in CRC compared to normal tissue and correlates with stage of disease	Pro-tumor	Not reported	(34)
	Overexpressed in CRC and associated with poor survival	Pro-tumor	Not reported	(35)
ATG7	Its deficiency induces metabolic defects and cell cycle arrest	Pro-tumor	Microbiome influenced immune response	(36)
	Its inhibition induced apoptosis, and it exerted synergistic effects with chemotherapy	Pro-tumor	LC3B	(37)
RACK1	Associated with increased autophagy in CRC, increased tumor aggressiveness	Pro-tumor	Phospho-JNK	(38)
KRAS	Gain of function activates autophagy in CRC cells to promote their survival	Pro-tumor	MEK/ERK pathway	(23)
SIRT1	Correlated with metastasis and invasiveness. Also, associated with cytoprotective autophagy after 5-FU treatment.	Pro-tumor	Fra-1; LC3	(39–43)
ATG10	Overexpression is associated with invasion and metastasis in CRC	Pro-tumor	Not reported	(44)
SphK1	Promotes autophagy-dependent invasion and metastasis	Pro-tumor	Paxillin	(45)
SOX2	Promotes malignant phenotype.	Pro-tumor	Beclin, β-catenin	(46)
Linc-POU3F3	Associated with an aggressive phenotype. Its inhibition induced cell cycle arrest and autophagy	Pro-tumor	SMAD4	(47)
PHLDA2	Promotes tumorigenesis. Its inhibition prevents EMT and increases autophagy	Pro-tumor	PI3K/AKT signaling pathway	(48)
RAMS11	Associated with aggressiveness. Its Inhibition prevented EMT and stimulated autophagy.	Pro-tumor	AKT/AMPKα/mTOR signaling pathways	(49)
LETM1	Associated with poor prognosis. Its inhibition induces autophagy which suppresses cancer stemness and proliferation	Pro-tumor	ROS-mediated AMPK/mTOR signalling	(50)
Beclin 1	Associated with overall survival, disease-free survival and favorable prognosis	Anti-tumor	Not reported	(51)
	Negatively associated with distant metastasis	Anti-tumor	Not reported	(52)
	Inhibited cancer cell growth and over expression enhanced effects of chemotherapy	Anti-tumor	Not reported	(53)
	Its loss is associated with poor clinical outcome.	Anti-tumor	Not reported	(54)
	Overexpression prevented invasion and migration	Anti-tumor	LC3-B and CDK4	(55)
LC3	Its loss is associated with poor clinical outcome	Anti-tumor	Not reported	(54)
ATG5	In Ras-mutated CRC cells knockdown of ATG 5 upregulated EMT markers	Anti-tumor	NF-κB pathway, p62	(56)
GRIM‐19	Down regulated in CRC patients. Suppress EMT and invasion through inhibition of hypoxia-dependent autophagy	Anti-tumor	STAT3/HIF-1α signaling	(57–59)
miR-140-5p	Weekly expressed in CRC. Reduces CRC progression and metastasis through suppression of autophagy	Anti-tumor	Smad 2 and ATG12	(60)
LACTB	Highly expressed in non-malignant CRC in comparison to malignant. Its inhibition prevents autophagy and promotes invasion and metastasis.	Anti-tumor	PI3K/AKT/mTOR pathway	(61)
miR-502	Is weakly expressed. It prevents cancer cell growth and autophagy.	Anti-tumor	Rab1B	(62)
UCA1	Inhibits autophagy and prevents CRC cell growth.	Anti-tumor	LC3	(63)
ROS	Activates mitochondrial signaling pathway and autophagic cell death in CRC	Anti-tumor	AMPK/mTOR pathway	(64)

A recent study demonstrated that CRC cell lines have a diverse sensitivity to autophagy modulators (65). Studies indicate that autophagy contributes to the early stage of CRC development, suggesting an important role of autophagy in CRC (36). It is now becoming clear that autophagy is involved in CRC development and progression, leading to a new insight into CRC therapeutic strategies using autophagy as a target. A study in CRC patients found LC3B expression is significantly co-related to aggressive tumor phenotype suggesting autophagy involvement in tumor promotion (28). One study showed that 38% of CRC patients had high LC3B levels and that LC3B was also significantly correlated with lymphatic invasion and p53 mutations (29). In regards to the CRC subtypes, the level of LC3B-II expression was higher in microsatellite stable (MSS) than that in MSI carcinomas (66). Immunohistochemistry of 68 patients with CRC revealed a significant correlation between Beclin 1 levels and those of LC3 and 4E-BP1 and overexpression of LC3 in tumor tissues compared to normal tissues (30). In another study done on 155 CRC patients’ overexpression of Beclin 1, a scaffold for the formation of autophagosomes was linked to tumor aggressiveness. However, patients with extensive over-or under-expression of Beclin 1 had a significantly poorer overall survival compared with the other two groups suggesting a dual role of Beclin 1 in CRC (31). In regards to the CRC subtypes, Wang et al., found that the expression of Beclin 1 and LC3 was unrelated to all clinicopathological parameters and overall survival in the MSI-H-CRC subgroup (67). Patients with colon cancer treated with adjuvant 5-FU who overexpressed Beclin 1 had a reduced survival rate, indicating autophagy’s role in chemoresistance (32). The inhibition of autophagy through genetic ablation of ATG5 inhibits tumor growth in colon cancer-derived cells and CRC patient-derived enteroid models (33). In a study involving patients and patient samples, LAMP3, a member of the family of autophagy-related proteins, is found to be higher in colorectal adenocarcinoma cells than in non-cancerous cells which is related to the stage of cancer (34, 35). In colon cancer samples, miR-502 expression was downregulated, suggesting it could serve as a tumor suppressor. Moreover, miR-502 inhibited autophagy in colon cancer cells suggesting that the effect of miR-502 has on colon cancer cell growth is partially due to the disruption of autophagy (62). The deficiency of ATG7 induced metabolic defects, an AMPK-mediated cell-cycle arrest, and AMPK activation in tumor cells but not in normal tissue (36). It was also found that ATG7 was crucial for the viability of CRC cells, and its inhibition induced cell death *via* activating apoptosis. Further, it exerted synergistic effects when combined with conventional chemotherapy (37). Both the autophagy markers, ATG5 and ATG 7, are known for their complex roles in other cancers (19); however, we found strong evidences supporting their pro-tumorigenic roles in CRC and targeting these genes could lead to enhanced antitumor responses (33, 36).

Survival rates were significantly higher for patients without ATG10-expressing tumors than those with ATG10-expressing tumors. Additionally, tumor invasion and metastasis are associated with the expression level of autophagy-related protein ATG10 (44). As colonic epithelial carcinogenesis progresses, RACK1 (receptor of activated kinase 1) increases remarkably and is positively associated with aggressiveness and negatively associated with patient survival. Further mechanistic analysis revealed that induction of autophagy by RACK1 led to proliferation and inhibition of apoptosis in colon cancer cells (38). Mutations in KRAS are known to be one of the most common causes of CRC (68, 69). In regards to the CRC subtypes, KRAS is found mutated in a substantial number of cases in CRCs, with a particular increased prevalence in MSS, however this prevalence decreases if only MSI CRC cases are studied (66). Through the MEK/ERK pathway, KRAS activating mutations increase autophagy in CRC cells, contributing to their survival under starvation conditions (23). This suggests that colorectal cancers resulting from KRAS mutations may require autophagy to survive. FOXO3A (Forkhead box O3), a member of the forkhead family of transcription factors, has been shown to activate apoptosis *via* upregulation of apoptotic genes and downregulation of antiapoptotic genes (70). In the absence of FOXO3A, cells may be more resistant to apoptosis and cell-cycle progression (71). In CRC cells, an increase in FOXO3A due to autophagy impairment caused an increase in transcription of a proapoptotic gene such as *BBC3/PUMA*, which resulted in apoptosis sensitization (72). In colon cancer cells, autophagy suppression enhanced apoptosis through p53 and UPR activation, resulting in antitumor effects (73). *In vitro* and *in vivo* findings in CRC cells suggest that autophagy inhibitors can significantly improve sinoporphyrin sodium-mediated photodynamic therapy-mediated anticancer activity (74). Hypoxia-induced autophagy plays a crucial role in the initiation and progression of CRC (75). Autophagy was found to be a mechanism through which long non-coding RNA urothelial carcinoma-associated 1 (UCA1) suppressed CRC cell growth. Autophagy inhibition by UCA1 suppresses cell proliferation and promotes apoptosis (63).

On the other hand, autophagy has been shown to reduce tumor development, supporting its tumor-suppressing role in cancer. Increased Beclin 1 expression was higher in CRC tissues than in normal tissues, and in CRCs, it was shown to be an important prognostic factor for overall survival and disease-free survival (51). In another study involving CRC patients, a negative correlation was seen between Beclin 1 protein expression and liver, whereas in distant metastasis there was no correlation with age, sex, depth of invasion, lymphatic or venous invasion, lymph node metastasis, tumor-node-metastasis staging, or differentiation (52). An additional study showed LC3 was suppressed in colorectal cancers along with a reduction in the expression of Beclin 1. Furthermore, *in vitro* experiments showed that overexpression of Beclin 1 inhibited CRC cell growth and further enhanced the antitumor potency of rapamycin (53). Interestingly, loss of various autophagy-related proteins such as Beclin1, LC3B and ATG5 is associated with poor clinical outcomes in CRC, and the prognostic impact of these proteins does not seem to be dependent upon already established clinicopathological parameters (54). The effects of silencing LETM1 (Leucine zipper-EF-hand-containing transmembrane protein 1), a protein overexpressed in CRC tissues compared to normal tissues were studied and were found to suppress cancer stemness and proliferation. Analysis of the autophagic response in cells revealed an increase in Beclin1 expression and a higher ratio of MAP-LC3II/I, suggesting an enhanced autophagic response after silencing LETM1 (50). A study found a down-regulation of ATG5 in CRC patients, but its increased expression was associated with lymph node infiltration (76). Using ATG5-deficient and Beclin1 knockout CRC cells, it was observed that the potency of niclosamide, an inhibitor of Wnt signaling was dependent on autophagy. Niclosamide inhibited mTORC1 and ULK1 activity and stimulated autophagy, making CRC cells sensitive to treatment (77). Through ROS/JNK signaling, photodynamic therapy induces autophagy-mediated cell death in human CRC, and knockdown of ATG5 or ATG7 inhibited the apoptosis of CRC cells by inhibiting the autophagic response after photodynamic therapy (78). In addition, it has been demonstrated that ERK inhibitors induce autophagy in CRC cells by activating ROS/p53 and cell death simultaneously (79). In CRC cells, hindering activated ROS-mediated mitochondrial signaling and AMPK/mTOR signaling pathways causes apoptosis and autophagy (64).

## Role of Autophagy in EMT and Metastasis

**Figure 2:**
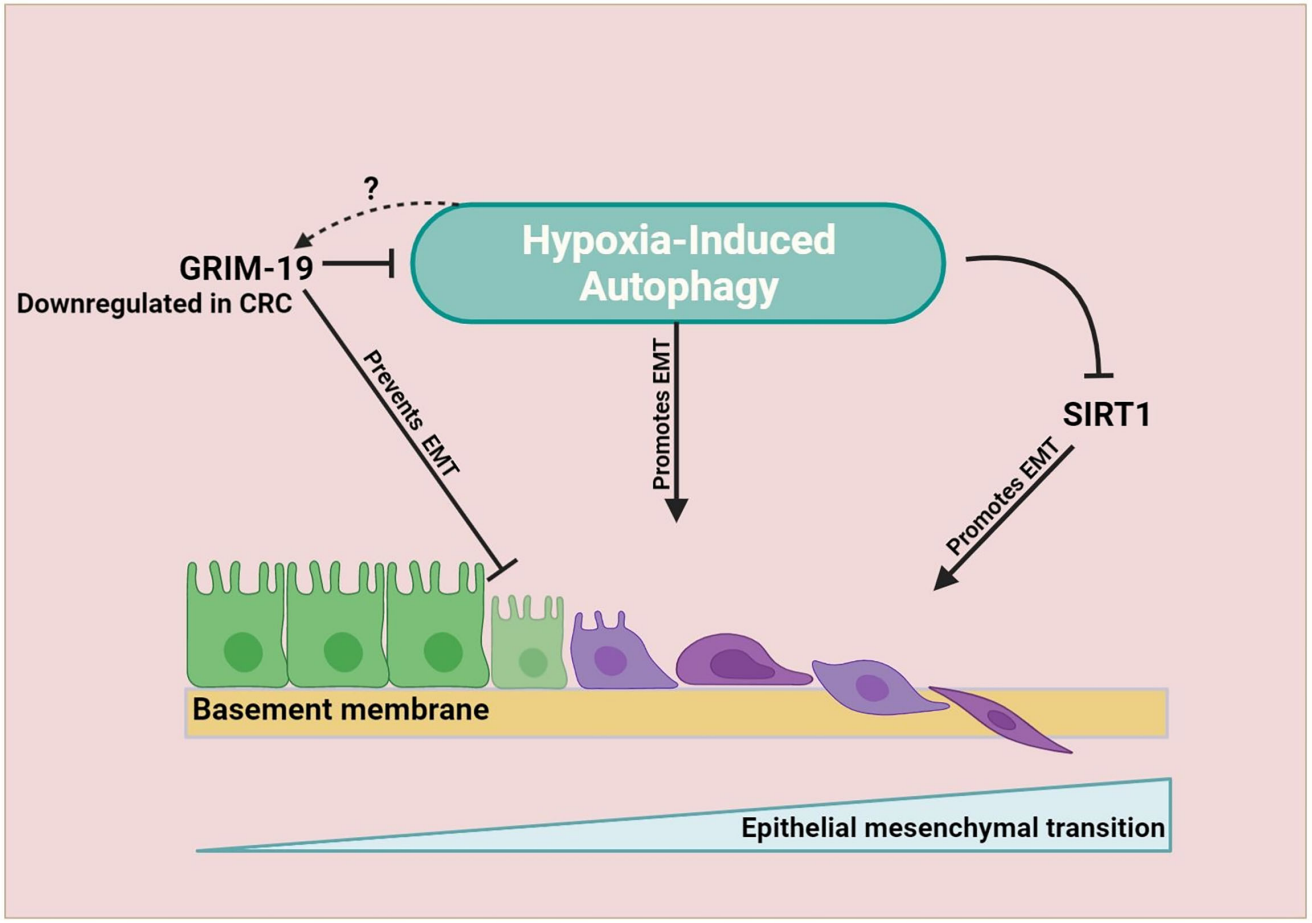
EMT is promoted by hypoxia-dependent autophagy in CRC. As the tumor progresses, hypoxia becomes more prevalent. A growing tumor triggers autophagy as an adaptive survival mechanism. Autophagy increases the plasticity of tumor cells, causing them to acquire mesenchymal characteristics. In CRC hypoxia-induced autophagy target SIRT1 promotes EMT process and hypoxia-induced autophagy inhibition by GRIM-19 prevents EMT. However, whether hypoxia-induced autophagy can upregulate itself by downregulating the expression of GRIM-19 is not known.

The epithelial-mesenchymal transformation (EMT) is a progressive process that allows epithelial cells to acquire mesenchymal characteristics. This gives epithelial cells properties such as motility and metastatic potential (80). Studies in recent years have shown that EMT plays a pivotal role in cancer cell invasion, metastasis, and drug resistance (81, 82). In this section, we will discuss recent evidence suggesting autophagy is involved in CRC cell plasticity and metastasis. EMT largely contributes to the pro-metastatic function of tumors, and studies indicate that there are several interactions between autophagy and EMT-associated signaling pathways. Consequently, inhibiting autophagy to control EMT development would be a promising strategy for CRC treatment (83). To date, the relationship between autophagy and EMT in tumors is not completely understood. Autophagy is involved in the metastasis of cancer cells as a double-edged sword. In the early stages, it inhibits cancer cells from spreading, but it promotes cancer cell survival and proliferation during advanced stages. During metastasis, cells must detach from the extracellular matrix, enter the bloodstream, and overcome environmental stresses such as hypoxia and nutrient deprivation. Due to the uncontrolled cell proliferation that outstrips the oxygen supply, tumors are gradually exposed to hypoxia, leading to tumor progression (84). It is well established that autophagy induction is one of the responses of cancer cells to hypoxia, and it is induced by hypoxia-inducible factor 1-alpha (HIF-1α). As hypoxia stimulates autophagy in cancer, blocking autophagy reduces the likelihood of metastatic and invasive progress (85). Hypoxia in CRC controls the EMT through various indirect mechanisms, for instance, gene associated with retinoid‐interferon‐induced mortality‐19 (GRIM‐19), a cell death regulatory protein known to act as a tumor suppressor (**Figure 2**) (86). GRIM‐19 expression was downregulated in CRC patients suggesting its favorable role in CRC carcinogenesis (57, 58). Also, it was shown to increase apoptosis, reduce invasion, and reduce migration in CRC (87). Recently GRIM‐19 was demonstrated to suppress EMT and the subsequent invasion through inhibition of hypoxia-dependent autophagy (59). However, it is not known if hypoxia-induced autophagy upregulates itself by degrading GRIM-19. Silent mating type information regulation 1 (SIRT1) an autophagy-related protein is associated with poor prognosis, tumor invasiveness, and metastasis in CRC patients (39–42). Recently, it was shown under hypoxic conditions SIRT1 modulates migration and invasion (88). Whether SIRT1-dependent autophagy has a role in CRC EMT is still not known.

ATG10 is an E2-like enzyme that is important for autophagosome formation. Its overexpression is associated with lymphovascular invasion and lymph node metastasis in CRC (44). Sphingosine kinase 1 (SphK1) is an enzyme that phosphorylates sphingosine to sphingosine-1-phosphate (SIP), which regulates proliferation and survival (89). Earlier, a study showed SphK1 modulated EMT markers in CRC and its inhibition reduced cancer cell migration (90). In support of this, a study published recently demonstrated that SphK1 stimulates autophagy in CRC cells and that autophagy driven by SphK1 may promote the invasion and metastasis of CRC by promoting the expression of focal adhesion paxillin (45). In another finding, CRC cells were protected from EMT by inhibiting autophagy. Through the inhibition of Beclin 1, expression of Twist and Vimentin (a mesenchymal marker) were decreased (91). Sex-determining region Y-box2 (SOX2) a transcriptional factor is associated with multidrug resistance genes and accelerated CRC progression (92). Recently it was found to promote malignant CRC phenotype through upregulated transcriptional activation of Beclin 1 (46). MicroRNA has-miR-140-5p was reported to be weakly expressed in metastatic tissues compared to normal mucosa. There was evidence that the hsa-miR-140-5p reduced CRC progression and metastasis through suppression of autophagy (60). Dysbiosis of gut microbiota is often associated with cancer (93, 94). Certain microbes can induce inflammation and promote tumorigenesis, such as *Fusobacterium nucleatum* (*F. nucleatum*). Recently it was reported that *F. nucleatum* is highly present in patients with metastatic CRC. Further investigations in mice and cell lines models revealed *F. nucleatum* infection upregulated autophagy and metastasis markers such as LC3-II, Beclin1, and Vimentin and lower levels of P62 and E-cadherin. Autophagy inhibitor treatment reversed the effects showing that *F. nucleatum* could promote metastasis in CRC *via* upregulation of autophagy (95).

In contrast, various studies reported the advantage of autophagy in protecting CRC cells against EMT and metastasis. In one study, autophagy inhibition in CRC cells was found to promote invasion and metastasis, which was further confirmed by an increase in metastasis markers such as N-cadherin, Snail family transcriptional repressor (SNAIl), and Twist Family BHLH Transcription Factor 1 (Twist1) proteins (96, 97). Similarly, in Ras-mutated HCT116 CRC cell line, a knockdown of ATG 5 upregulated EMT markers such as zinc finger box binding homeobox 1 (ZEB1), and SNAI2 (56). In addition to this, treatment of CRC cell lines with autophagy promotor Torin1 increased LC3 levels and downregulated Twist1 expression. Treatment with autophagy inhibitor compound MHY1485 increased p62 levels and upregulated the expression of Twist1 (98). Beta-catenin has a central role in directing several developmental processes. Wnt/β-catenin signaling pathway is crucial for various cellular functions and is abnormally activated in most colorectal cancers. Through the degradation of crucial mediators of Wnt signaling such as β-catenin, autophagy blocks EMT (99). A study found long intergenic noncoding RNAs (linc-POU3F3) control the CRC cell apoptosis, migration, and invasion *via* autophagy manipulation. Linc-POU3F3 is highly expressed in CRC and is correlated with tumor aggressiveness. Furthermore, *in vitro* studies showed inhibition of linc-POU3F3 induced cell cycle arrest and simultaneously increased autophagy (47). The mitochondrial serine protease Lactamase Beta (LACTB) is expressed in the CRC patient samples weakly, compared to the non-malignant CRC patient samples. Further *in vivo* and *in vitro* investigations demonstrated that LACTB knockdown inhibits autophagy as evident by lower LC3-II/LC3-I ratio, and induction of proliferation, invasion, migration, and EMT, whereas overexpression of LACTB has the opposite effect (61).

Autophagy activation was found to attenuate the migration of CRC cells (100). Yet another study reported that Beclin 1 overexpression prevented invasion and migration of aggressive CRC cells suggesting autophagy activation may reverse the aggressive cancers (55). Pleckstrin homology-like domain family A member 2 (PHLDA2) promotes tumorigenesis in CRC. PHLDA2 knockdown *in vivo* inhibits tumor aggressiveness and EMT and subsequently, increases autophagy (48). Long non-coding RNA RAMS11 was highly expressed in CRC cell lines and was associated with cancer aggressiveness. Further investigation showed that silencing of RAMS11 prevented EMT and stimulated autophagy *via* the mTOR-dependent pathway (49)

## Autophagy and Tumor Microenvironment

A solid tumor is highly heterogeneous at both cellular and acellular levels. The cellular components include mesenchymal cells, immune cells, and endothelial cells. On the other hand, the acellular components include extracellular matrix proteins, such as collagen, elastin, fibronectin, laminin, and secretory proteins, including cytokines, chemokines, proteases, growth factors, and metabolites (101). Cancer cells cause significant molecular, cellular, and physical changes in the surrounding tissues to promote tumor growth and progression. The TME is a complex and continuously evolving entity that differs between tumor types but is characterized by presence of immune cells, stromal cells, blood vessels, and extracellular matrix. It is proposed that the TME is not innocent during cancer progression but rather a decisive factor encouraging tumor growth. A potent complementarity emerges between cancer cells and cellular and acellular components of the TME during the early stages of tumor formation that aids tumor cell survival, invasion, and metastasis. Consequently, TME meshes a mechanism that promotes angiogenesis to restore oxygen and nutrient supply (102). In this section, we will discuss the main components of the TME, such as cancer-associated fibroblast cells, endothelial cells, and immune cells in the context of autophagy.

Cancer-associated fibroblasts (CAFs) are the most abundant types of mesenchymal cells in the tumor stroma that contribute to tumor initiation, immune evasion, and metastasis (103). According to a 2010 study, co-culturing CAFs with cancer cells activates hypoxia-induced autophagy in the CAFs, preventing apoptosis in cancer cells (104). Based on this, a model called “the autophagic tumor stroma model of cancer” was proposed. According to this model, autophagy activation in CAFs facilitates cancer cells’ survival and aggressiveness by providing them with recycled nutrients and building blocks. Nevertheless, another study showed that stromal fibroblast cells were essential for the metabolic reprogramming of CRC cells because they can induce oxidative stress in fibroblasts leading to changes in their metabolism. Oxidative stress and autophagy inhibitors could suppress the metabolic reprogramming between tumor cells and fibroblasts (105). Recently microRNA has been shown to play a pivotal role in cancer, exerting a regulatory effect on autophagy. In the development of CRC, miR-31 plays an important role in regulating autophagy by targeting various genes. Inhibition of miR-31 affects colon cancer by increasing autophagy in the CAFs of these cells, promoting proliferation, invasion, and metastasis in a co-culture system, as well as increasing cancer cells’ radiosensitivity (106).

Furthermore, loss of tumor protein p53, an oncogene, promotes the activation of surrounding fibroblasts into carcinoma-associated fibroblasts through the suppression of autophagy. It was found that exosome-derived miR-1434 from TP53-inactivated CRC cells interact with normal fibroblasts to suppress autophagy by targeting intracellular ATG2B, leading to fibroblast activation and induction of CRC cell proliferation (107). In contrast, another study demonstrated cancer-associated fibroblast-derived secretory mediator induced CRC cell proliferation, invasion, and migration by reducing autophagy (108). Several studies show that autophagy plays a role in tumor vasculature function. Endothelial cells in the adverse TME are deprived of nutrients resulting in hypoxia due to the restricted blood supply in solid tumors. This causes damage to tumor blood vessels, which become permeable and more fragile than normal blood vessels (109). There is mounting evidence suggesting that autophagy is important for endothelial cell homeostasis under physiological and pathological conditions, but whether autophagy plays a positive or negative role in the regulation of angiogenesis is still not clear (110, 111). It was observed that SIRT1 stimulates autophagy in tumor stroma under oxidative stress conditions, which aids endothelial cell survival (112). CRC-derived exosomes interfere with vascular endothelial barriers which stimulate angiogenesis and vascular permeability by transferring exosomes to the endothelial cells (113).

## Microbiota Induces Autophagy in CRC

Several studies have demonstrated that microorganisms may also contribute to the initiation and progression of cancer (114). Different types of cancer including CRC, are associated with microbial infections (115–119). Despite this, the correlation between cancer and microbiota is still unclear. As part of intestinal homeostasis, autophagy ensures intracellular defenses against microbes, maintains the integrity of secretory granules in Paneth cells, and mediates antigen presentation (120). Furthermore, mice models of CRC have demonstrated that gut microbiota plays a vital role in cancer development. It was found that about 80% of CRC cases are caused by the *APC* regulator of Wnt signaling pathway gene mutation (121). One of the best-known CRC models is Apc^Min/+^ mice bearing a loss-of-function mutation in the *Apc* gene (122). When compared with Apc^Min/+^ mice bearing microbiota, germ-free Apc^Min/+^ mice displayed a lower rate of intestinal and colorectal tumors (123). Microbiota in the gut is associated with cancer, particularly *F. nucleatum*. *F. nuclearum* induces chemoresistance by activating autophagy in CRC cells. *F. nucleatum* appears to be a driver of colorectal carcinogenesis since it is associated with a poor prognosis in CRC patients. By utilizing innate immune signaling and microRNAs, *F. nucleatum* altered chemotherapeutic responses for CRC (124). The crosstalk among microbiota and autophagy needs to be explored further to map the correlation better and to determine how they lead to cancer progression and whether probiotics could have potential to be used as a therapy to treat CRC remains an open question.

## Role of Autophagy in Immune Cells

As immune cells play a crucial role in the tumor microenvironment, an interesting paradox involves the relationship between immune cells and the tumor microenvironment, whereby immune cells can either suppress the growth of the tumor or promote it (125). Tumor response is regulated by both adaptive and innate immune cells. Innate immunity consists of non-specific responses involving macrophages, NK cells, neutrophils, and dendritic cells, while adaptive immunity is based on antigen-specific responses involving T and B cells. In the following subsections we will describe the effects of autophagy on innate and adaptive immune cells concerning CRC development and growth.

### Effects on NK Cells

The autophagy process has recently been linked to tumor immune evasion in pancreatic ductal adenocarcinoma. Natural killer (NK) cells have been considered the first-line defense against CRC since they can respond to stimulation within hours of stimulation, even without prior immunization (126, 127). Peripheral blood lymphocytes with medium to high cytotoxic activity have a lower cancer risk than those with low activity (128). Additionally, decreased NK cell frequency was associated with higher cancer risk and poor clinical outcomes in multiple cancers, including CRC (129). Numerous studies have found few NK cells infiltrating cancer cells or have impaired function (130–132). Dysfunctional NK cells often result from an imbalance of stimulatory and inhibitory receptors (133). In patients with CRC, NK cells isolated from PBMCs have expressed significantly lower levels of the activating receptor NKG2D (134). The role of NK cells in the progression of CRC has been supported by the finding that patients with CRC post-operatively develop lipid accumulation, which impairs their functions and facilitates the spreading of cancer (135).

Autophagy is involved in the differentiation and memory of NK cells, but how it modulates NK cell function in the CRC TME is not well understood. A recent study showed the importance of autophagy in mature NK cells development by ablation of autophagosome machinery component ATG5 in NK cells. The same study showed that NK cell’s autophagy promotes survival by limiting cell-intrinsic apoptosis. Further, treatment with metformin, a drug known to induce autophagy, increased lymphocyte counts by activating autophagy in an ATG5-dependent manner (136). According to a recent study, autophagy contributes to NK cell’s viability by removing damaged mitochondria and intracellular ROS. Further, the study reported that phosphorylated FoxO1 of NK cells interact with ATG7 on phagophores, causing autophagy and that FoxO1 deficiency undermines autophagy initiation which may impede NK cells development. This indicates that FoxO1-mediated autophagy is required for NK cells development (137). In support of this, another study showed that autophagy regulates NK cells’ survival and immunological memory. The same study showed that pharmacological activation of autophagy increased the number of memory NK cells through a mechanism that involves ATG3. This autophagy mechanism is important during the transition from effector cells to memory NK cells (138). The effect of autophagy activation and inhibition on NK cells infiltration into tumor growth sites remains largely unknown. However, limited evidence suggests inhibition of the autophagy protein Beclin1 in melanoma cells stimulates the infiltration of effector NK cells into the tumor bed through CCL5-dependent mechanism leading to the suppression of the tumor (139). *In vitro* studies demonstrated that breast cancer cells under hypoxic conditions could evade NK cells killing by enzymatic degradation of granzyme B (140). In contrast, in normoxia, when p53 function was restored to breast cancer cells, NK cells cytotoxicity is enhanced by p53 mediated autophagic sequestration of anti-apoptotic proteins facilitating Granzyme B-mediated mitochondrial outer membrane permeabilization or NK cells induced cell death (141). It is crucial to determine the effect of autophagy on tumor cells’ susceptibility to NK cells cytotoxicity *in vivo* as differences in the TME could alter the ability of autophagy to inhibit and/or enhance NK cells-induced apoptosis in tumor cells.

Together, these studies suggest that autophagy may play a role in the proper development of NK cells and that autophagic responses may modulate NK cell-dependent anticancer immunity. However, whether autophagy should be activated or inhibited in cancer treatment is a topic of extensive debate, and further studies are needed to determine its role in NK cells dependent anticancer immunity.

### Effects on Macrophages

Macrophages are an important member of the innate immune system since they phagocytose foreign substances and present them to the immune system. Activated macrophages are classified as M1 or M2 macrophages. M1 macrophages are primarily involved in pro-inflammatory responses, while M2 macrophages are predominantly anti-inflammatory (142). In tumors, M2 macrophages contribute to an immunosuppressive and tumor-promoting environment by secreting cytokines such as IL-10, IL-13, and TGF-β (143). The TME favors the M2 subset of macrophages through various mechanisms (144). It remains unclear what role tumor-associated macrophages (TAMs) play. Several studies have attempted to establish the association between macrophages density and CRC prognosis. Earlier research revealed a clear correlation between a high density of TAMs and a good prognosis in CRC (145–147). However, it is accepted that infiltration of M2 macrophages is associated with poor prognosis, metastasis, and tumor progression (148–151).

The results of one study demonstrated that autophagy was induced when monocytes were directed towards differentiation and that the induction of autophagy was crucial for monocytes’ differentiation into macrophages as well as for their survival (152). Additionally, inhibiting autophagy genetically or pharmacologically hinders differentiation, cytokine production, and induces apoptosis of monocytes, suggesting that autophagy is necessary for monocytes to macrophages differentiation (153). In addition, an *in vivo* and *in vitro* study demonstrated that pharmacological and genetic inhibition of autophagy prevented the differentiation of human monocytes into macrophages induced by colony-stimulating factor 1 (CSF-1) (153, 154). Yet another study involving ATG7 deficient macrophages reported that autophagy impairment caused reduced phagocytosis and respiratory burst activity. However, the same study found that macrophages could differentiate normally in the absence of autophagy (155). Another study reported that induction of mTOR-dependent autophagy was crucial for the proper differentiation of monocytes into macrophages (156).

The autophagy induction was also shown to be a critical regulator of the transition of TAMs to M2 phenotype. It was found that Chloroquine (CQ) treatment inhibits autophagy which in turn, impedes the transition of M1 into the M2 phenotype within TME (157). A study focused on the TME in colon cancer observed that co-culturing macrophages with colon cancer cells stimulated the release of epidermal growth factor (EGF) in CRC cells, which activated the EGFR/PI3K/AKT/mTOR signaling pathways, resulting in a polarization of TAMs into M2 phenotype (158). In a similar context, a study involving co-culturing macrophages with colon cancer cells demonstrated that autophagy manipulation in macrophages can affect colon cancer survival. The upregulation of autophagy in macrophages by autophagy-inducing treatments increased apoptosis in colon cancer cells and consequently, their sensitivity to radiotherapy (159).

Another study indicated that macrophages took up exosomes derived from CRC cells, which activated the PI3K/AKT pathway resulting in polarized M2 macrophages, and promoting liver metastasis (160). In addition, autophagy plays a crucial role in developing CRC in other innate immune cells, such as neutrophils. It has been found that tumor-associated neutrophils facilitate the progression of CRC, and that increasing neutrophil autophagy contributes to cancer cell migration (161, 162).

### Effects on Adaptive Immune Cells

In response to antigen stimulation, T cells differentiate into effector T cells, such as Th cells (Th1, Th2, Th17), Tregs, and cytotoxic T cells. Pro-inflammatory cytokines are produced by Th1, Th2, and Th17 cells, and anti-inflammatory cytokines are produced by Tregs. Cytotoxic T cells (CTLs) kill tumor cells by releasing perforin and granzymes (163). Over the past few years, several studies have demonstrated the importance of autophagy for T-cell activation, differentiation, and homeostasis (163–171). In this regard, autophagy has a crucial role in antigen processing and presentation to T cells (172). Autophagy has been reported to enhance the adaptive immune response by facilitating MHC I or MHC II-restricted presentation and maintaining T cells survival, function, and homeostasis (173). In response to TNF-α activation, autophagy was shown to improve the processing and presentation of mitochondrial viral antigens (174).

Two decades ago, Naito et al. showed that the infiltration of tumors with CD8^+^ T cells positively impacts CRC prognosis. CD8^+^ T-cells are widely recognized for their positive prognostic role in CRC (175–177). It has been reported that autophagy enhances the adaptive immune response by improving antigen-presenting cells (APCs) recognition, presentation, and maintaining T cell homeostasis in colitis-associated CRC (178). A recent study showed CD8^+^ based immune activity was increased by induction of autophagy in the intestinal epithelial cells. Increased autophagy causes lysosomal membrane permeabilization, enhancing MHC I presentation and CD8^+^ T cell activation (179). Autophagy was also essential for Treg cell’s survival, activity, and stability (180). As a result of the ablation of autophagy genes in Treg cells, fewer cells were produced, which led to an increase in the number of effectors CD8^+^ and CD4^+^ T cells infiltrating the tumor, resulting in increased tumor resistance (181). Another study showed that autophagy inhibition of T cells suppresses tumor growth in mice challenged with MC38 colon cancer and B16 melanoma cells (182). According to one study, the infiltration of Treg cells into CRC tumors is inversely correlated with the expression of autophagy-related protein SQSTM1 (183).

## Role of Autophagy in Resistance to Therapy

To prevent malignant transformation, autophagy ensures genome stability, removes abnormal organelles, and eliminates abnormal protein aggregation during the early stages of tumor formation. However, it also facilitates tumor progression by supporting tumor cell adaptation to the unwanted microenvironment. Upon chemotherapy radiotherapy or immunotherapy treatment, autophagy antagonizes, cooperates, or accompanies diverse cell death pathways.

### Role of Autophagy in Therapeutic Resistance

A growing body of evidence indicates that autophagy plays an integral role in developing chemoresistance. Additionally, various studies demonstrate that inhibition of autophagy combined with anticancer drugs can enhance cancer cell cytotoxicity and reduce resistance. To date, 5-fluorouracil (5-FU), along with other drugs like oxaliplatin, remains a widely used chemotherapeutic drug for treating CRC. Recently, we reported that 5-FU treatment upregulated LC3-II, SIRT1, and other autophagy proteins, inducing nucleophagy (a form of autophagy) in microsatellite stable (MSS) CRC cell lines, leading to resistance to 5-FU treatment (43). Nevertheless, multiple studies have shown autophagy induction after 5-FU treatment in CRC cells. It has been observed that mitogen-activated protein kinase 14 (MAPK14)/p38α helps protect colon cancer cells against the cytotoxic effects of 5-FU and irinotecan by triggering cytoprotective autophagy (184, 185). After 5-FU treatment, the autophagy response typically manifests itself as a pro-survival response in 5-FU-resistant cells, associated with higher activation of autophagy proteins. Using colon cancer cells in xenografts, it was found that curcumin enhances 5-FU antitumor effects by suppressing autophagic activity *via* AMPK/ULK1 signaling (186). Similarly, EGFR overexpression was shown to induce cytoprotective autophagy in CRC cells in response to 5-FU treatment (187). Ubiquitin-specific protease 11 (USP11) was found to induce resistance to 5-FU by inducing autophagy through AMPK/AKT/mTOR pathway in CRC cell lines (188).

Furthermore, microRNAs have been shown to play a role in chemotherapy sensitivity in CRC. It has recently been demonstrated that miR-125b confers resistance to 5-FU in CRC through induction of autophagy both *in vitro* and *in vivo* (189). Overexpression of miR-34a or knockdown of NEAT1 reduced the growth of CRC cell lines and made them more sensitive to 5-FU. Further studies found that NEAT1 facilitates autophagy in CRC cells by downregulating miR‐34a (190). MiR-34a modulates oxaliplatin resistance in CRC by enhancing macroautophagy *via* the TGF-β/Smad4 pathway. When miR-34a is inhibited, OXA is more effective at combating resistant CRC cells (191). MiR-22 also inhibits autophagy in CRC cells and promotes 5-FU induced apoptosis, killing the cancer cells more efficiently (192).

Additionally, CQ significantly enhances the anti-tumor effects of 5-FU both *in vitro* and *in vivo*. In an *in vivo* mice study, 5-FU induced autophagy was blocked by CQ treatment, which resulted in chemosensitivity (193). Similarly, in HT29 murine xenografts, sensitivity to oxaliplatin was augmented by autophagy inhibition with CQ treatment or by genetic manipulation of autophagy genes such as *Beclin 1* and *ATG5* (194). Metastatic CRC patients treated with HCQ (Hydroxychloroquine) demonstrate an improved immunity and a suppressed autophagy system by boosting p62 accumulation and upregulating lysosomal cathepsin D expression (195). The inhibition of autophagy by CQ increases the sensitivity of CRC cells *in vitro* to concurrent treatments with 5-FU and radiotherapy (196). Over the past few years, targeted therapy and immunotherapy-based treatment options have gained increasing popularity. This approach involves targeting molecular pathways crucial to tumor growth and maintenance, while immunotherapy involves stimulating the patient’s immune cells to detect and attack cancer cells. Recently, autophagy has been identified as a mechanism associated with cancer therapy resistance. Additionally, autophagy may modulate cancer immunotherapy response by degrading immune checkpoint proteins, releasing pro-inflammatory cytokines, generating and degrading antigens (197).

Specific therapies are available for patients with stage IV metastatic colon cancer these therapies target epidermal growth factor receptors (EGFR) such as cetuximab, Panitumumab, and angiogenesis such as cabozantinib, bevacizumab, and regorafenib. These drugs show improvement, but their effects are short-lived due to a rapid build-up of resistance (198, 199). Cetuximab works by blocking EGFR, thus inhibiting downstream EGFR signaling, negatively impacting tumor cell proliferation, invasion, and angiogenesis. Lately, it has been observed in an *in vitro* study that cetuximab treatment in many cancer cell lines, including CRC, stimulates cytoprotective autophagy through the inhibition of EGFR/PI3K/mTOR signaling pathway (200). An EGFR inhibitor, panitumumab, also inhibited cell proliferation in the DLD-1 CRC cell line, along with upregulation of autophagy genes, but no effect was seen on apoptosis and cell cycle progression, indicating that autophagy induction is responsible for reduced proliferation (201). Cabozantinib is a receptor tyrosine kinase inhibitor (TKI) that suppresses many targets, including VEGFR2. Recently, a study reported decrease in levels of activation PI3K/AKT/mTOR axis in colorectal cancer explants after cabozantinib treatment. Further *in vitro* investigation included cabozantinib combined with autophagy inhibitor treatment in CRC cell lines which resulted in the increased anticancer effect of cabozantinib (202). It is interesting to note that *in vitro* and *in vivo* studies showed that bevacizumab induces autophagy in CRC cell lines, as determined by the presence of increased autophagic markers. Additionally, inhibition of autophagy increases the cytotoxic effects of bevacizumab (203). Brigatinib a next-generationtion TKI, recently a study showed that brigatinib combined treatment with CQ enhanced the anticancer activity in CRC cells (204). Regorafenib is a multi-kinase inhibitor, and its resistance in CRC has been reported however the exact mechanism remains to be elucidated. Recently a study on glioblastoma found regorafenib treatment caused cytotoxicity through autophagy arrest (205). However, it remains to be determined if regorafenib combined with autophagy inhibitors would reverse the resistance in CRC.

In addition, a subgroup of CRCs with microsatellite instability (MSI) accounts for approximately 15% of all CRCs (206). Programmed cell death 1 (PD1) blocking antibodies that enhance tumor immunity are currently under investigation in MSI high CRCs (207). Immune checkpoints serve to prevent autoimmunity by modulating the immune system, but their presence becomes an escape mechanism for tumors from the immune system. CRCs with MMR/MSI-H respond well to PD-1 blockade immunotherapy (208). Nevertheless, it is estimated that 45-70% of these tumors showed resistance to immune checkpoint blockades (209–211). *In vitro* study involving RKO CRC cell line with BRAFV^600E^ and MSI-H phenotype treated with immune checkpoint inhibitors and anti-EGFR upregulated autophagy and PD-L1 levels *via* MEK/ERK signaling pathway. The inhibition of autophagy resulted in cells becoming more sensitive to immune checkpoint inhibitors and anti-EGFR treatments (212). Consequently, indicating autophagy induction as a mechanism of resistance to the immune checkpoint inhibitors. In addition, this suggests the need for *in vivo* studies to decipher how autophagy dysregulation might contribute to immune checkpoint inhibitor resistance in CRC patients.

## Conclusions

**Figure 3:**
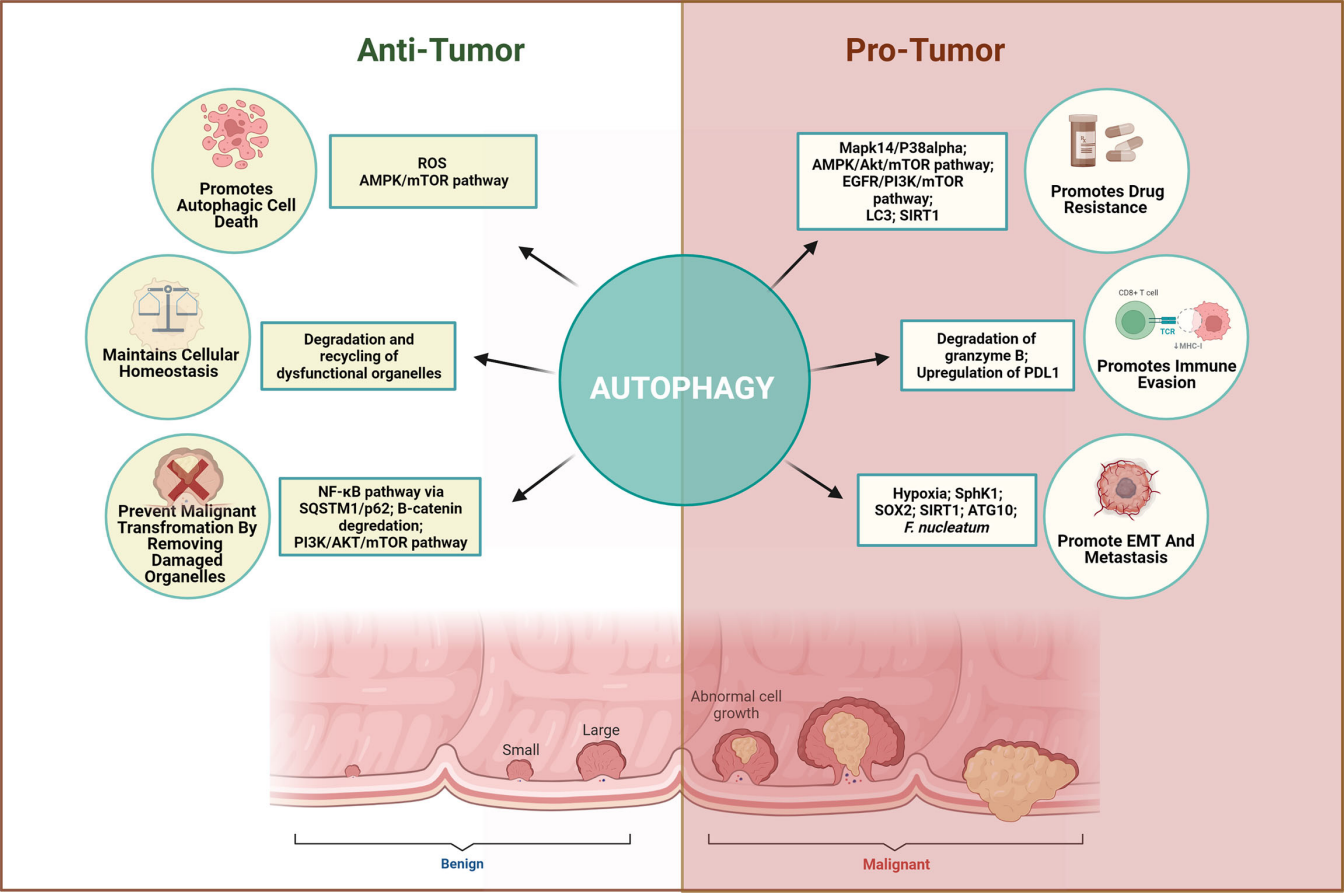
Dichotomous role of autophagy in CRC. Autophagy plays a complex and context-dependent role. On one hand, it can protect against abnormal survival by promoting autophagic death of tumor cells, maintain homeostasis and remove dysfunctional organelles in early stages, while on the other hand, it can promote tumor growth by favoring immune evasion, EMT, angiogenesis, and resisting the therapeutic effects when cancer has advanced.

Autophagy plays a complex and context-dependent role by limiting the cancer cell proliferation at the initial stages but facilitates cancer progression under stressful conditions in the later stages. Early retrospective studies using human CRC tissues have highlighted the link between autophagy and CRC. The majority of studies indicate that higher expression of autophagy proteins in CRC is associated with poor prognosis and metastasis. On the other hand, few studies have linked autophagy to a favorable prognosis in CRC. This discrepancy could be attributed to the different sizes of cohort and intra-tumoral genetic heterogeneity of the patients involved. Moreover, autophagy acts as an important switch in tumor progression and EMT. Hypoxia-dependent autophagy supports the cancer cell growth to support cancer cells in adverse conditions. Through regulation of autophagy, crosstalk among tumor microenvironment elements can influence tumor progression, EMT, and metastasis. To unravel mechanisms that control such a complex network, examining the autophagy network within the tumor microenvironment, including the CAF, immune cells, epithelial cells, and mesenchymal cells, should be advocated. **Figure 3** summarizes the dichotomous role of autophagy.

Furthermore, most studies examining the mechanistic role of autophagy on CRC tumorigenesis used xenograft mice models and CRC cell lines. Many of these studies have targeted autophagy transiently either through pharmacological or genetic manipulations, which can affect the study in various ways. Improved gene-editing techniques such as CRISPR/Cas9 are required to be used in preclinical research to delineate the mechanistic role of autophagy in CRC. In clinical trials, patients with mismatch repair deficiency or microsatellite instability-high CRCs responded better to immune-checkpoint inhibitors than CRC patients with MSS. However, most patients developed resistance during treatments. Currently, many clinical trials are underway utilizing autophagy inhibitors as an adjunct drug or in combinational chemotherapy (213). These trials intend to capture the positive outcomes of using autophagy as a process of inducing programmed cell death in cancer cells. Such autophagic manipulation may lead to promising effects in CRC treatment. Although there is ample evidence available and was discussed in this review, there are still many questions unanswered, especially when it comes to solving the paradox of autophagy switching from an anti-cancer to a pro-cancer mechanism. We believe that a comprehensive understanding provided by this study should clarify the gaps required to be filled by performing studies to effectively target autophagic pathways in combination with the conventional therapies in the treatment of CRC.

## Author Contributions

SM and JM were involved in the conception of the idea. SM wrote the initial draft prepared the tables and figures. QH, AM, and JM conducted supervision and critical review. SM, JM, AM, and QH revised and prepared the final version. All authors agreed to the final version of the manuscript.

## Conflict of Interest

The authors declare that the research was conducted in the absence of any commercial or financial relationships that could be construed as a potential conflict of interest.

## Publisher’s Note

All claims expressed in this article are solely those of the authors and do not necessarily represent those of their affiliated organizations, or those of the publisher, the editors and the reviewers. Any product that may be evaluated in this article, or claim that may be made by its manufacturer, is not guaranteed or endorsed by the publisher.

## Glossary

**Table:**

AMPK	AMP-activated protein kinase
APC	Adenomatous polyposis coli
APCs	Antigen-presenting cell
ATP	Adenosine triphosphate
BBC3	Bcl-2-binding component 3
BHLH	Basic helix–loop–helix
BRAFV600E	B-Raf proto-oncogene
CAFs	Cancer-associated fibroblasts
CCL5	Chemokine (C-C motif) ligand 5
CD4^+^	Cluster of differentiation 4
CD8^+^	Cluster of differentiation 8
CIMP	CpG Island Methylator Phenotype
CIN	Chromosomal Instability
CMA	Chaperon-mediated autophagy
CQ	Chloroquine
CRC	Colorectal cancer
CRISPR	Clustered Regularly Interspaced Short Palindromic Repeats
CSF-1	colony stimulating factor-1
CTLs	Cytotoxic T lymphocytes
EGF	Epidermal growth factor
EGFR	Epidermal growth factor receptors
EMT	Epithelial and mesenchymal transition
FIP200	Focal adhesion kinase family-kinase interacting protein of 200 kDa
FoxO1	Forkhead box protein O1
FOXO3A	Forkhead box O3
Fra-1	Fos-related antigen 1
GRIM‐19	Gene associated with retinoid‐interferon‐induced mortality‐19
HCQ	Hydroxychloroquine
HIF-1α	Hypoxia-inducible factor 1-alpha
HSC70	Heat shock cognate protein 70
IL-10	Interleukin-10
IL-13	Interleukin-13
JNK	c-Jun N-terminal kinase
KRAS	Kirsten rat sarcoma viral oncogene homolog
LACTB	Serine beta-lactamase-like protein
LAMP2	Lysosomal-associated membrane protein 2
LAMP3	Lysosomal Associated Membrane Protein 3
LC3	Microtubule-associated protein light chain 3
LC3B	Microtubule-Associated Protein 1 Light Chain 3B
LETM1	Leucine zipper-EF-hand-containing transmembrane protein 1
MAPK14	Mitogen-activated protein kinase 14
MAP-LC3II/I	Microtubule Associated Protein 1 Light Chain 3 II/I
MEK/ERK	Mitogen-activated protein kinase kinase/extracellular-signal-regulated kinase
MHC I	Major histocompatibility complex I
MHC II	Major histocompatibility complex II
miR-125b	MicroRNA-125b
miR-140-5p	MicroRNA 140-5p
miR-1434	MicroRNA-1434
miR-22	MicroRNA -22
miR-31	MicroRNA-31
miR‐34a	MicroRNA-34a
MMR	Mismatch repair
MSI-H	Microsatellite stability high
MSS	Microsatellite stable
mTORC1	Mammalian target of rapamycin complex 1
NEAT1	Nuclear Enriched Abundant Transcript 1
NKG2D	Natural Killer Group 2D
OXA	Oxaliplatin
p38αmapk	p38α Mitogen-Activated Protein Kinase
P53	Tumor protein P53
PBMCs	Peripheral blood mononuclear cell
PD1	Programmed cell death 1
PD-L1	Programmed death ligand-1
PE	Phosphatidylethanolamine
PHLDA2	Pleckstrin homology-like domain family A member 2
PI3K	Phosphatidylinositol 3-kinase
PI3P	Phospholipid 3-phosphate
PUMA	p53 upregulated modulator of apoptosis
RACK1	Receptor for Activated C Kinase 1
RAMS11	RNA associated with metastasis 11
ROS	Reactive oxygen species
SIP	Sphingosine-1-phosphate
SIRT1	Silent mating type information regulation 1
Smad4	Suppressor of Mothers against Decapentaplegic 4
SNAI2	Snail family transcriptional repressor 2
SNAIl	Snail family transcriptional repressor 1
SOX2	SRY-Box Transcription Factor 2
SphK1	Sphingosine kinase 1
SQSTMI	Sequestosome 1
TAMs	Tumor-associated macrophages
TGF-β	Transforming growth factor β
Th Cells	T helper cells
Th1	T helper 1 cells
Th17	T helper 17 cells
Th2	T helper 2 cells
TKI	Tyrosine kinase inhibitor
TME	Tumor microenvironment
TNF-α	Tumor Necrosis Factor alpha
UCA1	Urothelial carcinoma-associated 1
ULK1	Unc-51 Like Autophagy Activating Kinase 1
ULK2	Unc-51 Like Autophagy Activating Kinase 2
UPR	Unfolded protein response
USP11	Ubiquitin-specific protease 11
VEGFR2	Vascular Endothelial Growth Factor Receptor-2
VPS15	Vacuolar protein sorting 15
VPS34	Vacuolar protein sorting 34
WIPI1	WD repeat domain, phosphoinositide interacting 1
WIPI2	WD repeat domain, phosphoinositide interacting 2
ZEB1	Zinc finger E-box binding homeobox 1
4E-BP1	Eukaryotic translation initiation factor 4E (eIF4E)-binding protein 1
5-FU	5-fluorouracil
